# TNO1, a TGN-localized SNARE-interacting protein, modulates root skewing in *Arabidopsis thaliana*

**DOI:** 10.1186/s12870-017-1024-4

**Published:** 2017-04-11

**Authors:** Rahul Roy, Diane C. Bassham

**Affiliations:** 1 Department of Genetics, Development and Cell Biology, 1035B Roy J Carver Co-Lab, 1111 WOI Rd, Iowa State University, Ames, IA 50011 USA; 2 Interdepartmental Genetics Program, Iowa State University, Ames, IA USA; 3 Plant Sciences Institute, Iowa State University, Ames, IA USA; 4 Current Address: Department of Plant and Microbial Biology, University of Minnesota, Twin Cities, MN 55108 USA

**Keywords:** *t*rans-Golgi network, Root movement, Cell file rotation, Tethering factor, Microtubules

## Abstract

**Background:**

The movement of plant roots within the soil is key to their ability to interact with the environment and maximize anchorage and nutrient acquisition. Directional growth of roots occurs by a combination of sensing external cues, hormonal signaling and cytoskeletal changes in the root cells. Roots growing on slanted, impenetrable growth medium display a characteristic waving and skewing, and mutants with deviations in these phenotypes assist in identifying genes required for root movement. Our study identifies a role for a *trans*-Golgi network-localized protein in root skewing.

**Results:**

We found that *Arabidopsis thaliana* TNO1 (TGN-localized SYP41-interacting protein), a putative tethering factor localized at the *trans*-Golgi network, affects root skewing. *tno1* knockout mutants display enhanced root skewing and epidermal cell file rotation. Skewing of *tno1* roots increases upon microtubule stabilization, but is insensitive to microtubule destabilization. Microtubule destabilization leads to severe defects in cell morphology in *tno1* seedlings. Microtubule array orientation is unaffected in the mutant roots, suggesting that the increase in cell file rotation is independent of the orientation of microtubule arrays.

**Conclusions:**

We conclude that TNO1 modulates root skewing in a mechanism that is dependent on microtubules but is not linked to disruption of the orientation of microtubule arrays. In addition, TNO1 is required for maintenance of cell morphology in mature regions of roots and the base of hypocotyls. The TGN-localized SNARE machinery might therefore be important for appropriate epidermal cell file rotation and cell expansion during root growth.

## Background

Establishment of the root system of a plant is crucial for efficient anchorage and nutrient acquisition. Root development and architecture are well-studied [1] but our understanding of how roots interact with soil components is still incomplete. This is due in part to the difficulty of visualizing roots as they move into the soil, although recent developments such as the GLO-Roots platform aid in imaging this process [2, 3]. Root growth into the substratum presents multiple cues to the root tip such as mechanical obstacles, moisture and nutrient gradients. These cues are integrated and then signal downstream processes involving hormonal pathways and cell expansion [4]. This leads to a cumulative physiological response driving root movement and establishing the root architecture.

Studying and simulating root movement and directional growth in the laboratory on synthetic growth media has revealed distinct movement types, and some of the genes and pathways that control them [4]. Roots of *Arabidopsis thaliana* seedlings display various growth behaviors depending on external conditions. When embedded in a homogeneous medium (penetrable agar), roots grow downward in response to gravity and show minimal deviation from the gravity vector. By contrast, when roots are subjected to multiple directional cues, complicated growth patterns occur [5]. For example, *Arabidopsis* roots grown on a slanted impenetrable medium (1.5% agar) show a characteristic deviation from the vertical (skewing) with a periodic wave-like pattern along their trajectory (waving). This arises due to a combination of touch [6], gravitropism [7], circumnutation [5], and physical interaction between the root tip and the growth medium [8].

Skewing and waving roots display a characteristic twisting of epidermal cell files along the root, referred to as cell file rotation (CFR). When roots of *Arabidopsis* seedlings skew and wave, the succession of sinusoidal waves alternate between left-handed and right-handed CFRs, which correlates with their rightward and leftward movement respectively [6, 7]. According to Rutherford and Masson (1996), skewing is described as rightward or leftward when viewed from the back of the plate [7] while handedness of the CFR is defined as left-handed or right-handed when viewing the axis of the root pointing shootwards. Roots of *Arabidopsis* grown on a slanted, hard medium exhibit a dominant left-handed rotation around the growth axis resulting in a predominantly counterclockwise/left-handed epidermal CFR. This usually corresponds to a rightward skewing when seen from the back of the plate [7, 9, 10]. CFR also typically correlates with an oblique microtubule (MT) array orientation in the twisting cell files, although exceptions do exist [11].

Multiple factors such as external cues (e.g. moisture, light or gravity), hormonal pathways, and cytoskeletal and cell wall dynamics influence the direction of root growth [4]. External cues signal changes in hormone signaling pathways, including auxin [12–14], ethylene [8, 15], cytokinin [16] and brassinosteroid pathways [17]. Downstream of hormonal and environmental perception, changes in the cytoskeleton and in cell wall deposition patterns modulate cell division and cell expansion dynamics, thus mediating root movements. Defects in tubulin structure or activity or in MT-associated proteins result in altered MT dynamics and array orientation. This then leads to changes in cell expansion and affects CFR and skewing [18, 19]. Cell wall properties and the trafficking of cell wall components to the plasma membrane are key to root elongation and movement as inferred from the altered root movements observed in mutants defective in cellulose deposition, wall-anchored proteins or crosslinking of cell wall components [20–22].

Sorting of cell wall components occurs at the *trans*-Golgi network (TGN), a tubulo-vesicular organelle that matures from the two or three *trans*- most cisternae of the Golgi [23]. It acts as an early endosome, receiving endocytosed cargo as well as directing vacuolar and secretory traffic, with distinct subdomains that function in various trafficking routes [24–30]. Specific membrane fusion factors called SNAREs (soluble *N*-ethylmaleimide–sensitive factor attachment protein receptors) maintain vesicle trafficking fidelity and cargo sorting within cells [31]. The TGN-localized SYP4 (41/42/43) SNARE family is required for multiple transport pathways that in turn regulate auxin homeostasis and disease resistance [30]. SYP61, another TGN-localized SNARE that interacts with SYP41, functions in vacuolar trafficking and secretion of cell wall enzymes such as cellulose synthase and pectin-polysaccharide-modifying protein. This indicates a role for SYP61 in trafficking of cell wall components and enzymes for their synthesis and modification [32–34]. TNO1 (TGN-localized SYP41-interacting protein) is a TGN-localized coiled-coil protein that interacts with the SYP41 SNARE machinery. Mutant plants lacking TNO1 (*tno1*) partially mis-sort vacuolar cargo, mis-localize SYP61 and display altered TGN dynamics and slower gravitropic responses [35, 36].

Trafficking at the TGN plays a role in auxin responses, trafficking of cell wall components, and cell expansion, all of which are important for root growth and movement. In addition, gravitropic bending is delayed in *tno1* roots, suggesting defects in directional growth of roots. We therefore investigated a potential function for TNO1 in root movement. We report here that TNO1 acts as a negative regulator of root skewing, since *tno1* mutant roots have enhanced skewing which also correlates with an enhanced CFR.

## Methods

### Plant material and growth conditions

*Arabidopsis thaliana* Col-0 (wild-type, WT) and *tno1* knockout mutant (SALK_112503) were obtained from the Arabidopsis Biological Resource Center; the complemented *tno1* mutant was generated previously in our laboratory [35]. *Arabidopsis* seeds were surface-sterilized in 33% bleach, 0.1% (*v*/v) Triton X-100 for 20 min, rinsed 5 times with sterile water and kept in the dark at 4 °C for at least 2 days before being subjected to the root skewing and waving assays or drug sensitivity assays described below. *Arabidopsis* hypocotyls were analyzed by plating sterilized seeds on 0.5X solid Murashige-Skoog (MS) medium [(Murashige-Skoog vitamin and salt mixture, Caisson, MSPA0910] with 1% sucrose, 2.4 mM MES (pH 5.7), and 0.8% (*w*/*v*) Phytoblend agar (Caisson, PTP01) and growing vertically at 22 °C in the dark.

### Root skewing and waving assays

Skewing and waving assays were performed as described [7, 37]. *Arabidopsis* plants were grown at 22 °C in long-day conditions (16 h light) on 0.5X solid Murashige-Skoog (MS) medium (Murashige-Skoog vitamin and salt mixture, Caisson, MSPA0910) with 1% sucrose, 2.4 mM MES (pH 5.7), and 1.5% (*w*/*v*) Phytoblend agar (Caisson, PTP01). Seedlings were grown vertically for 3 days after which the root tip position was marked. The plates were then slanted backwards, 30^o^ to the vertical, and grown for another 4 days. Images of the roots were acquired from the back of the plate using a Nikon SMZ1000 light microscope equipped with a Nikon S10 CoolPix camera. Analysis of root parameters was performed using Image J [38]. Root tip deviations to the right from the back of the plate were considered positive while deviations to the left were considered negative.

Root morphometric analyses were performed as described [39]. Images of skewing/waving roots were analyzed with the ImageJ software [38] for measuring root tip abscissa (Lx) and length of the root (L) followed by calculation of horizontal growth indices (Lx/L).

### Drug sensitivity assays

Seedlings were grown vertically on plates containing either taxol (Paclitaxel, Sigma Cat #T7191) or propyzamide (Sigma-Aldrich, Cat #45645) at the indicated concentrations for 5 days and skewing angles of roots were determined. Dark grown hypocotyl cell phenotypes were analyzed by placing 7-day-old hypocotyls on the surface of warm 3% low melting point agarose on a slide [40] and then imaging the imprints with a Zeiss Axioplan II light microscope.

### Cell file rotation analysis and propidium iodide staining

The root surface of *Arabidopsis* seedlings was visualized using a Zeiss macro-zoom microscope at the Microscopy and NanoImaging facility, Iowa State University. Alternatively, roots were stained with propidium iodide (ThermoFisher Scientific Cat #P3566). Seedlings were dipped in a working aqueous solution (10 μg/ml) of propidium iodide for 1 min and then washed twice by dipping in petri dishes filled with water for 30 s. The roots were mounted in water and visualized using a Leica confocal laser scanning microscope (Leica SP5; Leica Microsystems) at the Iowa State University Confocal and Multiphoton Facility. Excitation and emission wavelengths were 488 nm and 617 nm respectively. Laser power, scan frequency and line averaging were optimized and kept constant between samples and replicates. CFR angles of the roots were calculated as described [7, 41] using ImageJ [38].

### Immunostaining and analysis of microtubules

Five-day-old *Arabidopsis* seedlings grown on slanted medium were fixed and immunostained as described [42]. Mouse anti-α**-** tubulin antibodies (Sigma-Aldrich, Cat #T6074), diluted 1:100, were used for immunolabeling of MTs followed by AlexaFluor 488-conjugated goat anti-mouse IgG (ThermoFisher Scientific, Cat #A11029) as a secondary antibody, diluted 1:500. Cells within the elongation zone were imaged by confocal microscopy with a Leica confocal laser scanning microscope (Leica SP5; Leica Microsystems) at the Iowa State University Confocal and Multiphoton Facility, using a 40X (1.25 NA) or 63X (1.4 NA) Leica oil immersion objective. Excitation and emission wavelengths were 488 nm and 507 nm. Laser power, scan frequency and line averaging were optimized and kept constant between samples and replicates. Confocal images were analyzed with the freely available software package MicroFilament Analyzer (MFA) [43], and data was displayed as circular plots as generated by the software. MT angles generated by the software were classified into three classes, 0 to 10, 11 to 30 and 31 to 90 degrees using Microsoft Excel and used for subsequent analysis.

## Results

### Loss of TNO1 results in exaggerated root skewing

Mutants lacking TNO1 have normal root elongation, but show a lag in root gravitropic bending upon gravistimulation [36]. Positive gravitropism is a key component driving root waving and skewing, and hence we hypothesized that TNO1 might play a role in these processes.

**Fig. 1:**
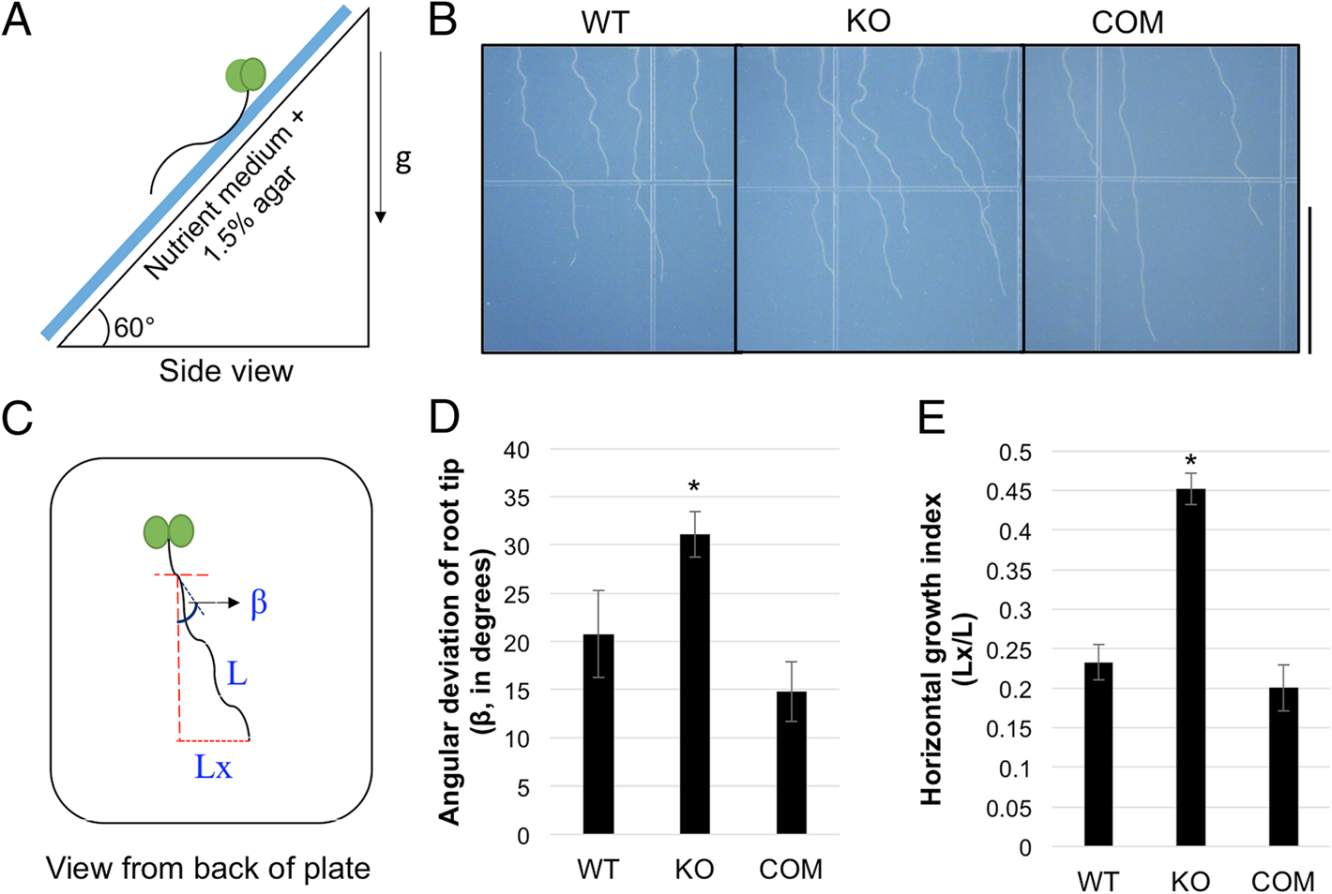
Loss of TNO1 protein function causes increased rightward root skewing. **a** Side view of the setup for the root skewing assay on growth medium solidified with 1.5% agar. Seedlings were grown vertically for 3 days in long day (LD) conditions, after which the root tip position was marked. The plates were then slanted backwards, 30^o^ to the vertical, and grown for another 4 days. **b** Images of WT, *tno1* mutant (KO) and complemented *tno1* mutants (COM) displaying skewing of roots away from the vertical. Scale bar =1 cm. **c** Schematic diagram of analysis of a root to determine the angular deviation of the root tip (β), the horizontal root-tip deviation (Lx) and the length of the root (L). A higher value of β and HGI (=Lx/L) indicate increased skewing. **d** Angular deviation of root tips of WT, KO and COM seedlings subjected to the root skewing assay as described in (a). **e** Horizontal growth index (HGI) of roots of WT, KO and COM seedlings subjected to the skewing assay. HGI is the ratio of the horizontal root tip deviation (Lx) and root length (L). All values represent the means of 3 biological replicates with 12–15 seedlings analyzed for each replicate. Error bars indicate standard error. *Asterisks* indicate a statistically significant difference (*P* < 0.05) by Student’s *t*-test

To test this hypothesis, 3-day-old seedlings were grown vertically on the surface of hard agar medium (1.5% agar), the plates tipped over by 30^o^, and the seedlings allowed to grow for an additional 4 days (Fig. 1a). The slant forces the root tip to press against the impenetrable medium due to positive gravitropism. As the root fails to penetrate the medium, cell expansion and circumnutation drive the root to the side and this alternates between rightward and leftward corrective deviations resulting in characteristic waving and skewing responses [4, 7]. *tno1* mutant roots (KO) formed sinusoidal wave-like growth patterns similar to WT (Col-0) roots but had an exaggerated rightward skew compared to the WT roots, when seen from the back of the plate (Fig. 1b). *tno1* mutants complemented with transgenic *TNO1* under the control of its own promoter (COM) [35] had reduced skewing compared to the mutants and were similar to the WT roots (Fig. 1b). Root images were analyzed using ImageJ [38] and quantified for deviation of the root tips from their position at the time of slant initiation (β) (Fig. 1c) [7, 39]. *tno1* mutants had a higher angle of deviation than the WT or complemented roots (Fig. 1d; *P* < 0.05). To confirm the difference in phenotype, another morphometric parameter, the horizontal growth index (HGI), was calculated for the skewing roots. HGI is the ratio of the root tip abscissa (Lx) and length of the root (L) (Fig. 1c) and is a sensitive and robust parameter to quantify lateral directions of growth, independent of root shape [39]. Root tips skewed to the right yield positive Lx values and negative values when roots skew left. A positive Lx results in a positive HGI value, which then signifies a rightward deviation while a negative value suggests leftward deviation [39]. *tno1* roots had a significantly higher HGI than WT and complemented lines (Fig. 1e; *P* < 0.05), confirming that the *tno1* mutants do indeed have an increased rightward deviation from the vertical.

### Skewing *tno1* roots display enhanced root epidermal cell file rotation (CFR)

The cellular basis for CFR is still not completely understood, but has been proposed to be a result of the circumnutation of the root tip [20]. Circumnutation is dependent on an internal mechanism that results in an elliptical or circular trajectory around an imaginary axis of growth [44]. The helical pattern of cell division at the root tip has been hypothesized to be the basis for epidermal CFR [6, 10, 37]. The characteristic spiral cell division patterns of the outer circle of meristematic cells in the root apex causes a spiraling of the cell files, thus resulting in CFR formation [45], although this fails to explain why CFR initiates at the base of the elongation zone, away from the site of initial cell divisions. A lag in anisotropic expansion rates between the epidermal and the inner cell layers can also cause a twisting of the cell files to compensate for stresses and strains that might otherwise cause tissue breakage [9]. CFR, usually preceding root bending and skewing [41], is visible at the base of the elongation zone of skewing roots. Rightward-skewing mutants usually display a dominant left-handed CFR [4, 11]. We therefore hypothesized that the enhanced rightward skewing in the *tno1* mutants would correlate with an increase in left-handed CFR.

**Fig. 2:**
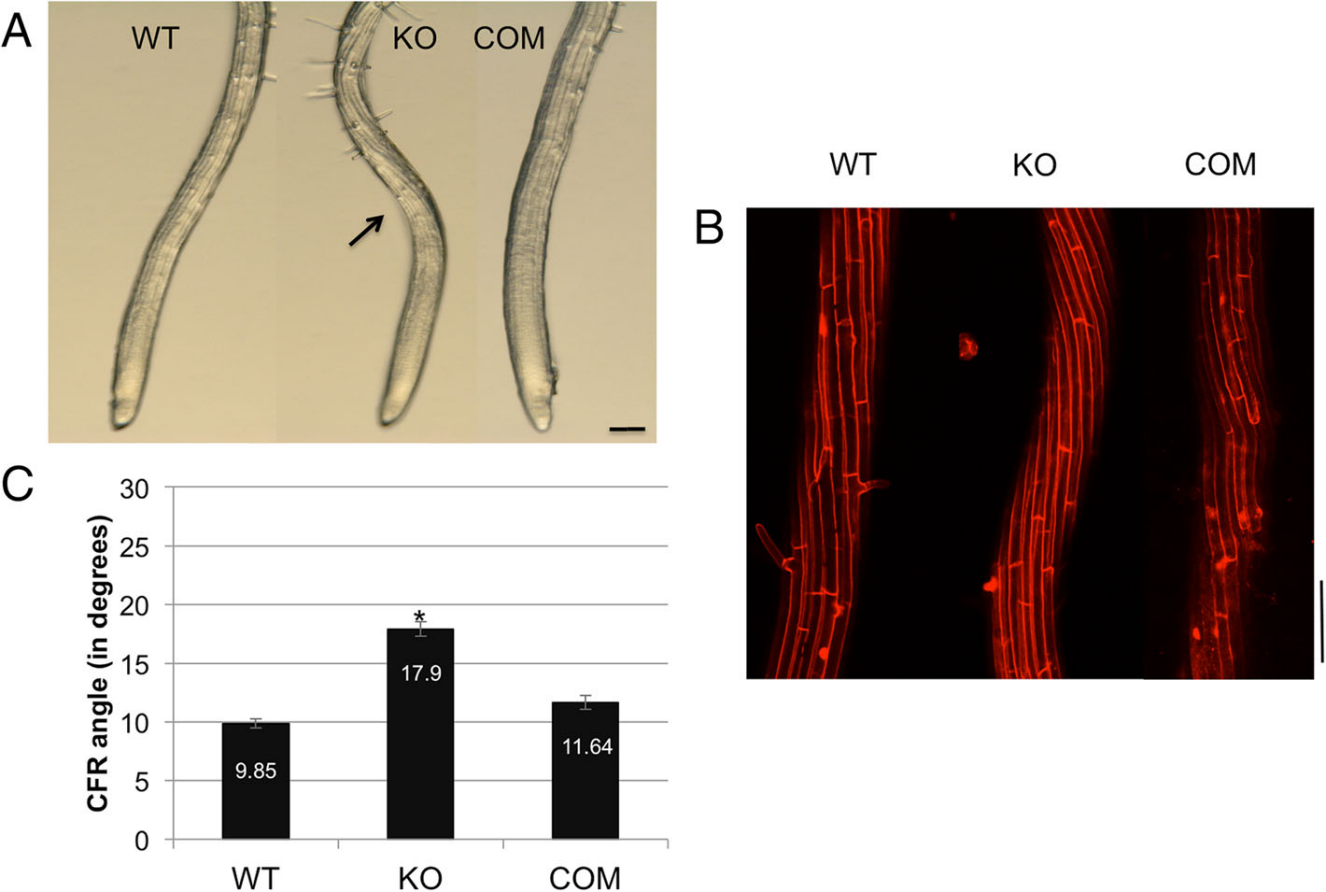
Skewing *tno1* roots display enhanced root epidermal cell file rotation. **a** Images of the surface of root tips of WT, KO and COM lines subjected to the skewing assay, imaged under a macro zoom microscope. The *black arrow* points to epidermal cell file rotation. Scale bar =100 μm. **b** Confocal microscopic image of propidium iodide-stained elongation zone of WT, KO and COM roots subjected to the skewing assay. Scale bar =100 μm. **c** Cell file rotation angles of skewing roots of WT, KO and COM lines. All values represent the means of 3 biological replicates, with at least 10 cell files from 5 seedlings analyzed for each replicate. Error bars indicate standard error. *Asterisks* indicate a statistically significant difference (*P* < 0.01) by Student’s *t*-test

Microscopic analysis of mutant roots showed a marked left-handed epidermal CFR initiating in the root elongation zone (black arrow, Fig. 2a) while WT and complemented roots mainly have a vertical arrangement of cell files with a lack of distinct CFR in this region (Fig. 2a). Confocal imaging of propidium iodide-stained roots revealed a distinctive left-handed CFR with a higher twist in the elongation zone of *tno1* lines compared to the WT and complemented lines (Fig. 2b). Images were analyzed with ImageJ to quantify the left-handed twist of the cell files relative to the longitudinal axis of the root. The *tno1* CFRs were significantly higher (*P* < 0.01) than the WT and complemented root CFRs (Fig. 2c). This may explain the enhanced skew of the mutant roots, since the increased left-handed twist might cause larger deviations from the axis of growth.

### Skewing of *tno1* roots is enhanced upon MT stabilization by taxol

CFR can be caused directly by altered cortical MT organization, which influences anisotropic expansion as MT alignment controls the direction of cellulose deposition [46, 47]. Several mutants defective in MT stability, organization and dynamics have enhanced right- or leftward skewing, with a dominant left or right-handed CFR respectively [18]. Many, but not all, also display a handedness of the cortical MT arrays that is opposite to the CFR handedness of the elongation zone of the roots. Some mutants defective in MT-interacting proteins show right-handed CFR, skew to the left, and display left-handed helical cortical MT arrays in the elongation zone [48, 49], while others have skewing defects but normal transverse MT array orientation in the root elongation zone. This suggests that the relationship between MT dynamics, orientation, and skewing is complex. We hypothesized that the enhanced skewing and CFR in *tno1* mutants might also be due to changes in cortical MT array organization or dynamics. TNO1 is localized to the TGN, and this hypothesis receives support from a recent study in which a TGN-localized protein was shown to activate a kinesin and be involved in root skewing and gravitropism, linking MTs and the TGN in the root skewing process [50].

**Fig. 3:**
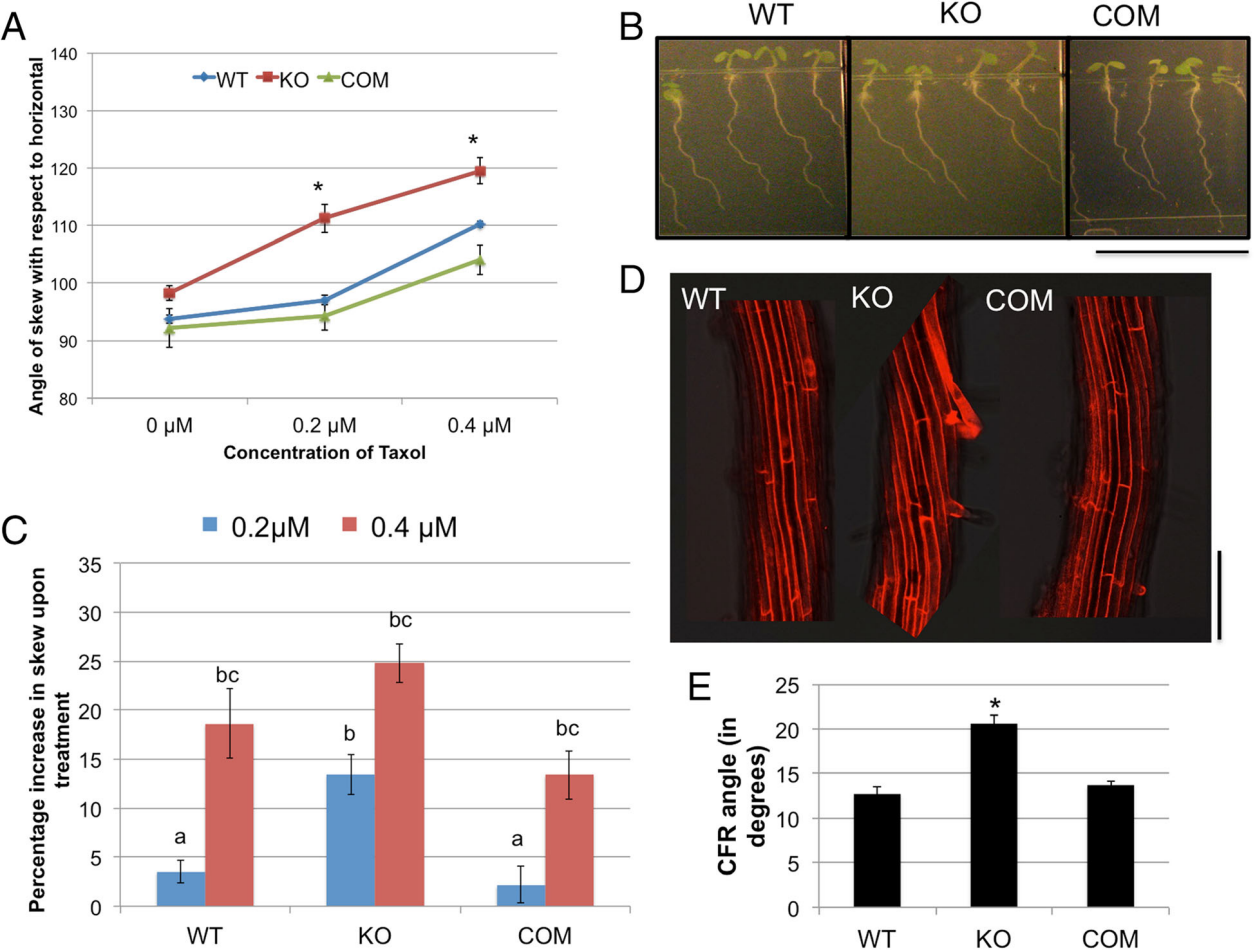
Skewing of *tno1* roots is enhanced upon MT stabilization by taxol. **a** Angle of skew with respect to the horizontal for WT, KO and COM roots grown on two different concentrations of taxol. Seedlings were grown vertically in LD conditions for 5 days on growth media solidified with 1.5% agar and either containing taxol or solvent as a control. All values represent the means of three biological replicates with at least 12–15 seedlings for each replicate. Error bars indicate standard error. *Asterisks* indicate a statistically significant difference (*P* < 0.05) by Student’s *t*-test. **b** Light microscopic images of roots of WT, KO and COM lines grown vertically in the presence of 0.2 μM taxol for 5 days. Scale bar =1 cm. **c** Mean root skew of each genotype on taxol compared to the mean on the solvent control was expressed as percentage increase in skew. Values represent analysis of 3 biological replicates with at least 12 seedlings for each replicate. Error bars indicate standard errors. Different letters indicate statistically significant differences (*P* < 0.05) by Student’s *t*-test. **d** Confocal images of propidium iodide-stained elongation zones of WT, KO and COM roots displaying epidermal CFR. The roots were grown vertically on 0.2 μM taxol for 5 days. Scale bar = 100 μm. **e** Cell file rotation angles of skewing roots of WT, KO and COM lines grown on 0.2 μM taxol. All values represent the means of 3 biological replicates with at least 10 cell files from 3 seedlings analyzed for each replicate. Error bars indicate standard errors. *Asterisks* indicate a statistically significant difference (*P* < 0.01) by Student’s *t*-test

The MT-stabilizing chemical taxol causes MT bundling and enhances rightward skewing in WT roots [9, 51]. When grown vertically on increasing concentrations of taxol (Fig. 3a), *tno1* roots showed significantly higher rightward skewing than the WT and complemented lines (Fig. 3a, b). The percentage increase in the skewing angle of the mutant roots from 0 μM to 0.2 μM taxol is significantly higher (*P* < 0.05) than in the WT and complemented line roots (Fig. 3c). We hypothesized that this increase in skewing was due to an increase in the left-handed twist of the roots. Analysis of confocal images of propidium iodide stained roots in the presence of 0.2 μM taxol reveals a larger left-handed CFR in the *tno1* roots compared to WT and complemented lines (Fig. 3d, e). The phenotype is reflective of the effect of taxol on the root growth rather than the effect of a slanted substratum, since the drug assay is conducted on vertically-oriented plates. These results suggest that MT stabilization exaggerates the root skewing and CFR phenotype of the *tno1* mutants.

### Effect of MT destabilization by propyzamide (PPD) on root skewing in *tno1* mutants

**Fig. 4:**
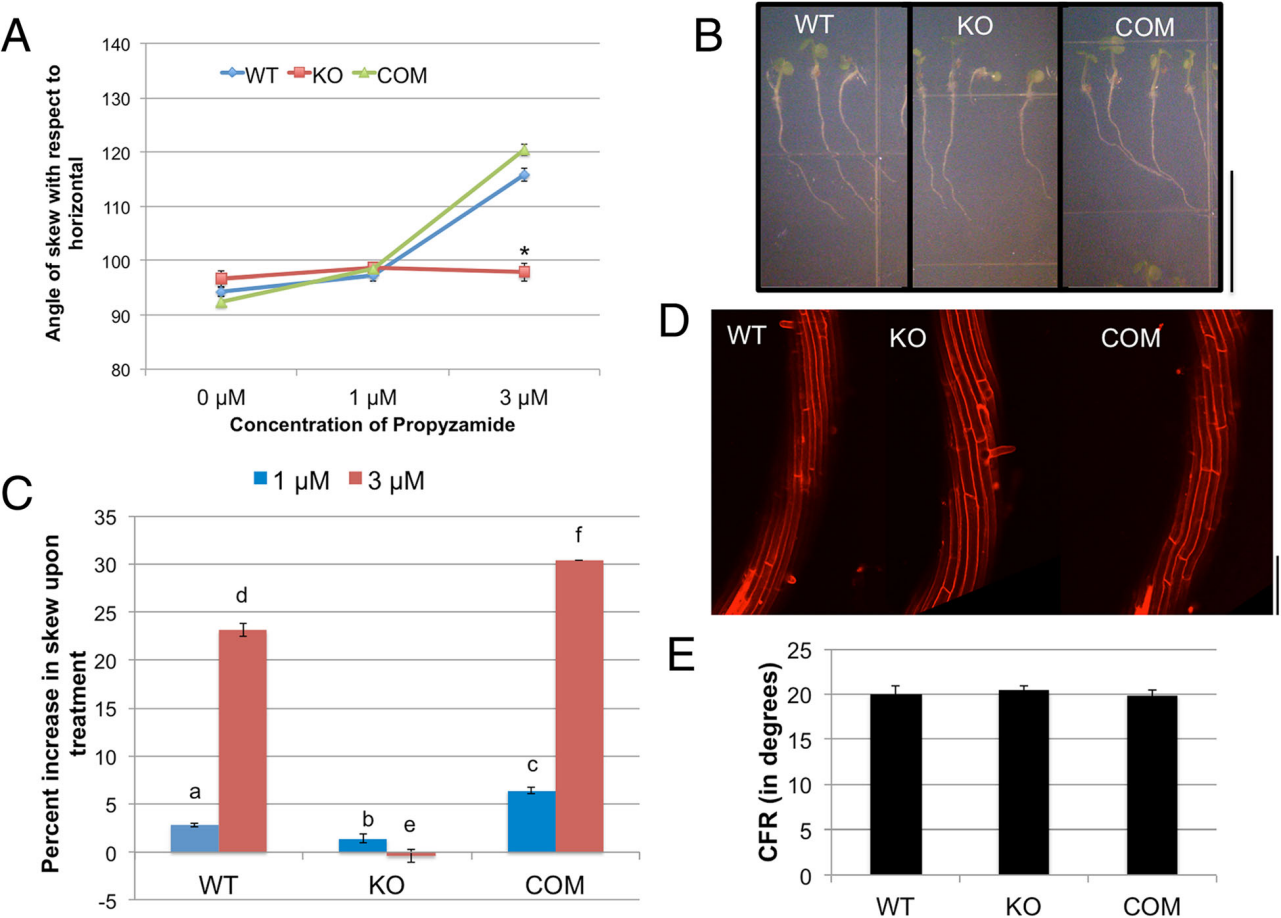
Effect of MT destabilization by propyzamide (PPD) on root skewing in *tno1* mutants. **a** Angle of skew with respect to the horizontal for WT, KO and COM roots grown on two different concentrations of PPD. Seedlings were grown vertically in long-day conditions for 5 days on growth media solidified with 1.5% agar and either containing PPD or solvent as a control. All values represent the means of 3 biological replicates with at least 12–15 seedlings for each replicate. Error bars indicate standard error. *Asterisks* indicate a statistically significant difference from WT (*P* < 0.05) by Student’s *t*-test. **b** Light microscopic image of roots of WT, KO and COM lines grown vertically in the presence of 3 μM PPD for 5 days. Scale bar =1 cm. **c** Mean root skew of each genotype on PPD compared to the mean on the solvent control, expressed as percentage inhibition. Values represent analysis of 3 biological replicates with at least 12 seedlings for each set. Error bars indicate standard error. Different letters indicate statistically significant differences (*P* < 0.05) by Student’s *t*-test. **d** Representative image of propidium iodide-stained elongation zones of WT, KO and COM roots grown vertically on 3 μM PPD for 5 days, imaged with a confocal microscope. Scale bar = 100 μm. **e** Cell file rotation angles of roots of WT, KO and COM lines grown on 3 μM PPD. All values represent the means of 3 biological replicates with at least 10 cell files from 3 seedlings analyzed for each replicate. Error bars indicate standard errors

The MT-destabilizing chemical propyzamide (PPD) also enhances rightward skewing in WT seedlings [9]. Since the effect of MT stabilization on root skewing in *tno1* seedlings suggests a MT-based role for TNO1 in root movement, we tested the effect of PPD on root skewing in the *tno1* mutant. WT and complemented roots showed a slight increase in rightward skew at 1 μM PPD and a marked increase at 3 μM PPD. Surprisingly, skewing of *tno1* mutant roots was insensitive to PPD (Fig. 4a, b). The percentage increase in skewing observed for WT and complemented roots in the presence of PPD was significantly higher than in *tno1* (Fig. 4c). The increase in skewing for the complemented lines was slightly higher than the WT, suggesting that the precise expression pattern of the TNO1 protein may be reflected in the extent of root skewing. While the TNO1 transgene in the complemented lines is expressed under its native promoter and its overall expression levels are similar to that of the endogenous protein, position effects within the genome may cause slight differences in expression pattern in different cell types.

We hypothesized that the reduced skewing of *tno1* roots in the presence of PPD would correlate with a reduced CFR when compared with CFRs of PPD treated-WT and complemented roots. Confocal microscopic analysis of the surface of roots growing on 3 μM PPD reveals a twisting of WT and complemented epidermal cell files. The mutant root epidermal cell files also display considerable left-handed CFR, indistinguishable from WT and complemented lines (Fig. 4d, e). Hence, the resistance of *tno1* roots to PPD-induced skewing is independent of PPD-induced CFR. To explain this observation, we hypothesized that the PPD treatment might have other effects on the mutant roots, including effects on root growth or morphology. There were no measurable differences between the root growth rates of the WT, *tno1* and complemented lines when grown in the presence or absence of PPD (not shown), and it is therefore unlikely that the difference in skewing is due to differences in growth rate.

**Fig. 5:**
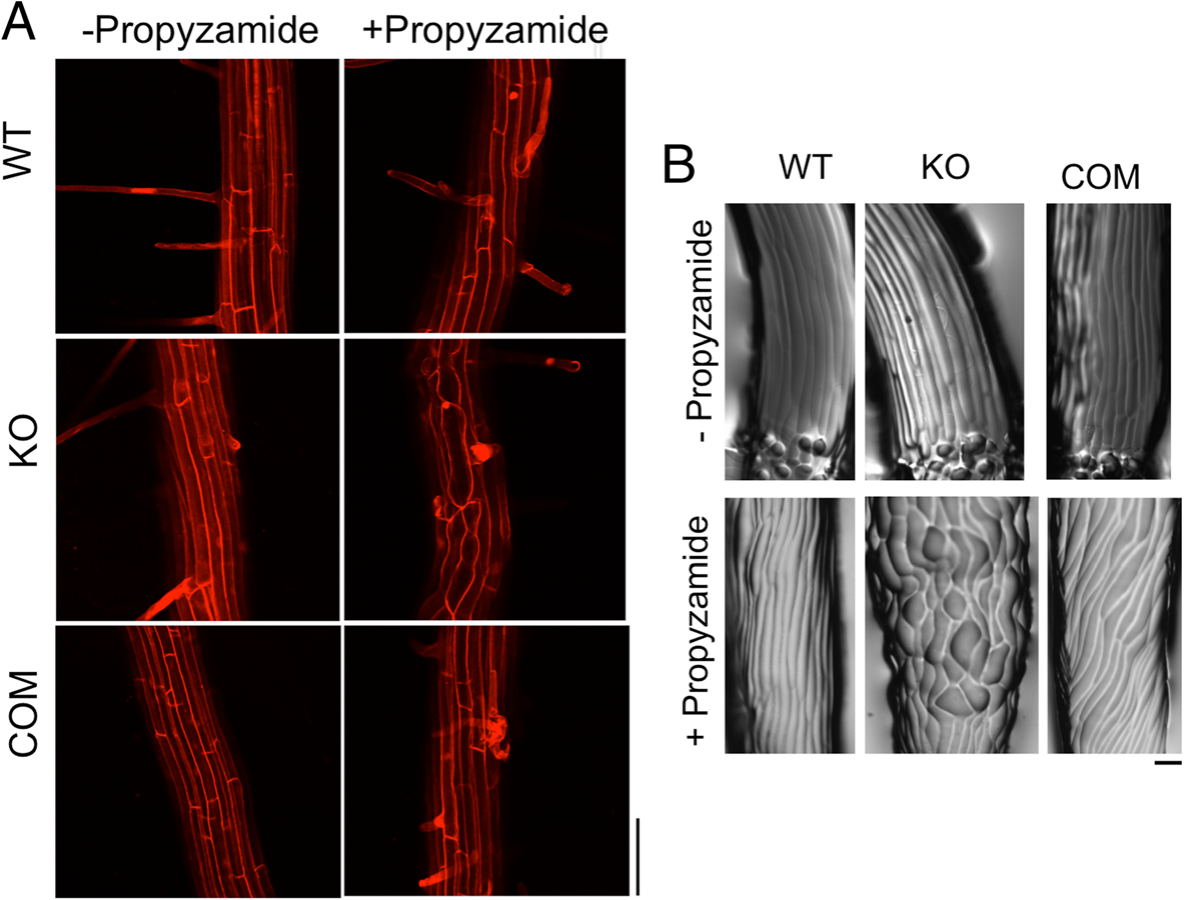
Effect of 3 μM propyzamide (PPD) on cell morphology of the mature region of the root and the base of the hypocotyl. **a** Representative image of propidium iodide-stained mature zone of WT, KO and COM roots grown on solvent as a control or 3 μM PPD for 7 days, imaged with a confocal microscope. Scale bar =100 μm. **b** Representative agarose imprints of the base of WT, KO and COM hypocotyls grown on solvent control or 3 μM PPD for 7 days, imaged with a macro zoom microscope. Scale bar =100 μm

Surprisingly, mature regions of *tno1* roots grown on 3 μM PPD had more severe defects in cell morphology when compared to PPD-treated WT and complemented lines, with the mutants displaying malformed cells (Fig. 5a). Whereas PPD-treated WT and complemented roots have typical long, narrow cells, *tno1* mutant root cells are shorter and wider, indicating defects in anisotropic growth. This points to a possible defect in cell maturation or cell wall deposition that could potentially interfere with the ability of the *tno1* roots to skew in the presence of PPD even as the CFR in the elongation zone continues to form normally. It is possible that the defects in the mature zone of the PPD-treated *tno1* roots could then affect how the growing root interacts with the substrate, which would in turn affect root skewing.

To assess whether other organs and cell types in the mutant might also be sensitive to MT disruption by PPD, we analyzed agarose imprints of control and PPD- treated dark-grown hypocotyls. The base of the hypocotyls in the WT and complemented lines had cells with increased width when compared to their untreated counterparts. *tno1* hypocotyls were hypersensitive to PPD, with much more severe defects in cell morphology when compared to the untreated *tno1* seedlings and to PPD-treated WT and complemented seedlings (Fig. 5b). The PPD-treated *tno1* seedlings also lacked the spiralization of the cells observed in the treated WT and complemented lines. As observed in the skewing assay, the complemented lines were slightly more sensitive to PPD than WT, which again may be due to position effects. Cell elongation is initiated at the base of dark grown hypocotyls [52], and the loss of TNO1 may lead to an increased susceptibility of this cell expansion process to MT destabilization by PPD. Treatment with low concentrations of PPD (such as 2 μM) has been reported to cause a decrease in the dynamic instability of cortical MTs, leading to more static MTs that spend more time in the paused state compared to untreated cells [53]. Our results suggest that the disruption of MT dynamics by PPD has a more adverse effect on *tno1* roots than WT. This could be manifest as defects in MT-array organization, anisotropic expansion, or both, resulting in the observed disruption in cell morphology in *tno1*.

### The orientation of MT arrays in the elongating cells of skewing *tno1* roots is not affected

MT arrays in elongation zones of roots are usually arranged in a transverse orientation to facilitate anisotropic expansion. Skewing roots of some mutants with marked CFRs have distinct oblique arrays of MTs, with the handedness of a MT array opposite to that of CFR [18]. Strong correlations have also been observed between angle of skewing and pitch of the MT arrays [11]. Many mutants that are defective in MT-associated processes have a change in the pitch of MT arrays associated with a corresponding change in CFR. However, some mutants with enhanced CFR show no obliqueness of the MT array, but rather have transverse arrangements similar to the central elongation zone cells of the WT roots. For example, the *sku6* mutant maintains a transverse alignment of MT arrays despite its left-handed CFR [9, 54] while the *mor1–1* mutant, impaired in MT polymerization, also displays a left-handed CFR but no bias in MT array orientation [55]. These mutant phenotypes suggest that additional factors can also be responsible for a change in CFR, apart from the MT array orientation.

**Fig. 6:**
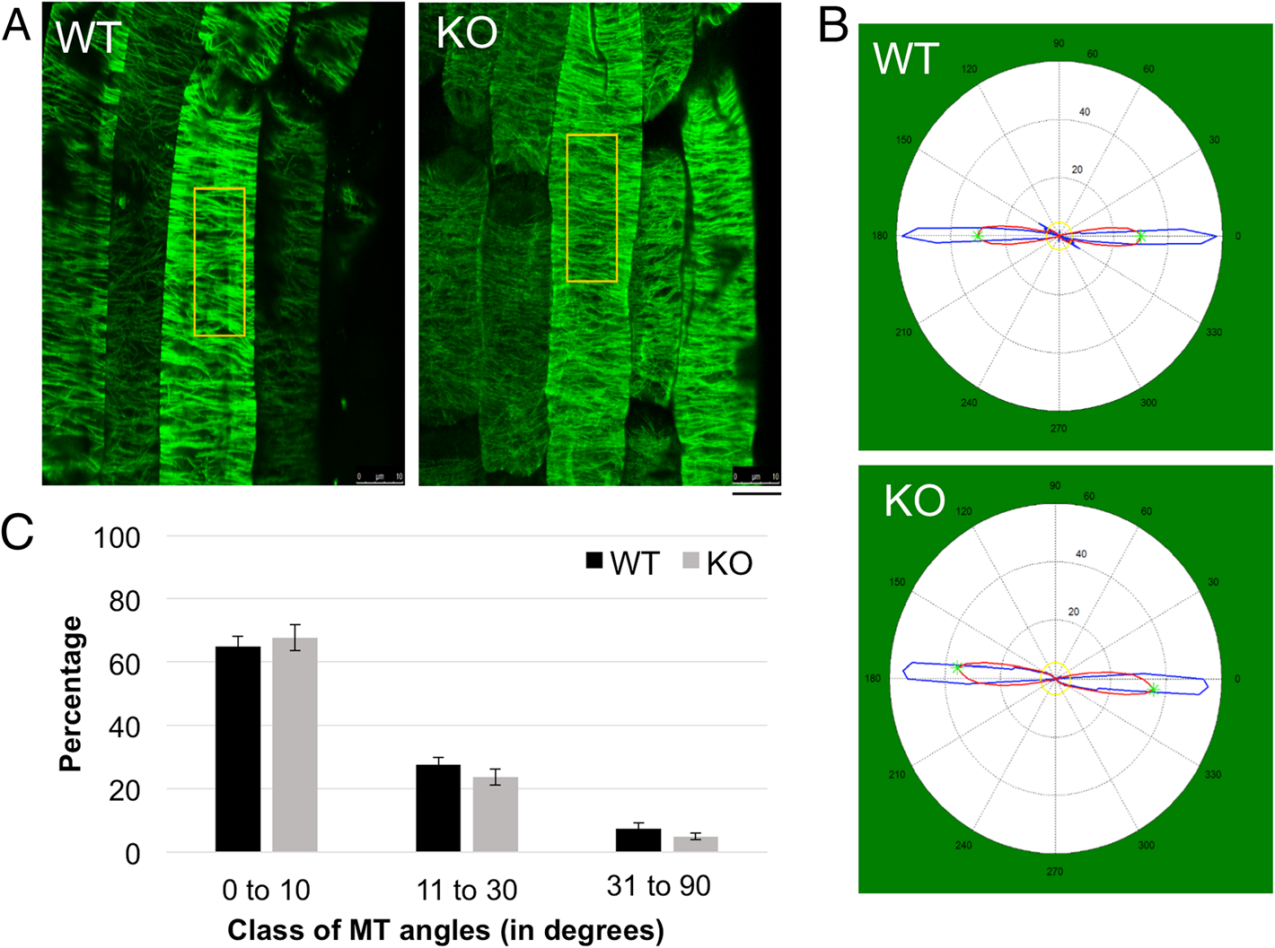
The orientation of MT arrays in the elongating cells of skewing mutant roots is not affected. **a** Representative confocal images of the cortical microtubule array of elongating cells of 5-day-old *Arabidopsis* roots grown on slanted medium, immunostained with anti-α**-** tubulin. The boxed inset indicates the region that was selected for the MT array analysis in 6b. Scale bar =10 μm. **b** Graphical representation of the analyzed MTs as a circular plot derived from analysis of the confocal images in part a with the MicroFilament Analyzer software (MFA). The blue line represents the original data (the MT angles) while the red line represents smoothed data. Green asterisks denote dominant orientations while the yellow line is a weighted average of the detected filaments per angle. **c** Microtubule angle distribution in the elongating cells of WT and KO roots was calculated from the data generated via the MFA software. Values represent analysis of 3 biological replicates with at least 500 MTs analyzed from 3 cells of three different roots for each replicate. Error bars indicate standard errors

We hypothesized that the dominant left-handed CFR in the *tno1* roots would correspond to a right-handed MT array in the epidermal cells of the elongation zone compared to WT roots. Four-day-old chemically fixed WT and *tno1* roots, grown on a slanted impenetrable medium, were immunostained with tubulin antibodies [42] to detect MTs and imaged by confocal microscopy. The MT arrays of epidermal cells in the mutant roots did not show any distinct handedness or change in pitch compared to WT epidermal MT arrays (Fig. 6a). Quantitative analyses on collected images were performed utilizing the MATLAB software package MicroFilament Analyzer [43] to allow comparison of MT orientation in multiple images from different biological replicates. The representative circular output graphs from the analyses suggest a mainly transverse orientation of the MTs in the elongation zone of both WT and *tno1* roots (blue line along the 0–180 degree axis) (Fig. 6b). To assess whether a difference in the percentage of oblique MTs between the WT and mutant might explain the higher CFR in the mutants, MT angles of root cells from WT and *tno1* roots were classified into three classes, 0 to 10, 11 to 30 and 31 to 90 degrees (Fig. 6c). No significant difference was found in the percentage of MTs in each class between the WT and mutant. Thus we conclude that *tno1* mutants have normal transverse MT arrays in the skewing roots, suggesting that an alternative mechanism must be responsible for the change in CFR.

## Discussion

TNO1 plays important roles in post-Golgi trafficking, gravitropism and auxin transport-dependent processes. Here we demonstrate that TNO1 is required for root skewing and CFR formation. Skewing of *tno1* mutant roots increased to a greater extent upon MT stabilization by taxol when compared to WT roots but was resistant to MT destabilization by PPD. This led us to postulate a role for TNO1 in MT-dependent processes, although MT destabilizing agents have also been reported to induce left-handed CFR without affecting MT arrays [53]. In roots, MT arrays transition from a disordered state in the meristematic zone to a transverse orientation in the elongation zone, and this organization is important for facilitating rapid anisotropic growth [42]. Defects in tubulin structure/activity and in MT-associated proteins can result in oblique MT arrays in the central elongation zone of roots. Oblique MT orientation in elongating cells causes cells to expand at an angle to the vertical, leading to CFR and root skewing [4, 20, 56, 57]. Skewing roots of *tno1* do not show an oblique MT orientation in the elongation zone but rather a transverse one. This suggests that the root movement phenotype in *tno1* mutants arises independent of the organization of MT arrays in the root elongation zone, although dependent on MT dynamics. It is possible that, despite its normal orientation, the cortical MT array in *tno1* roots might be compromised in restructuring or function as the root navigates a complex trajectory during skewing. The loss of TNO1 could potentially also affect the function of a MT-associated protein such as a kinesin; as an example, a TGN-localized kinesin-binding separase has recently been reported to affect root skewing [50].

Certain root skewing mutants have transverse MT arrays or a lack of directional bias in the MT arrays in the root elongation zone but have a distinct CFR [54, 58, 59]. Moreover, CFR can still occur upon MT disruption with MT destabilizing agents such as oryzalin and PPD [53]. This suggests that the MT array is not the only factor involved in generating CFR. Owing to anomalies such as these, Wasteneys (2004) proposed that CFR could arise due to torsional handedness inherent to roots, and the correlation between CFR and MT array orientations could result from signaling between the MT array and cell wall machinery. Thus, CFR could arise from the effect of MT arrays on anisotropic expansion in the elongation zone or, alternatively, from the effect of cellulose microfibrils on MT array alignment at the plasma membrane [46, 47]. Mutants that affect CFR formation but not MT array orientation in the elongation zone support this model, including mutants defective in regulation of anisotropic cell expansion [20, 54]. Based on this model, and consistent with the cell morphology phenotypes observed in the presence of PPD, TNO1 might be important for the anisotropic cell expansion process or in cell wall trafficking dynamics.

A lag in the expansion rates of internal cell types compared to the epidermal cells may cause roots to skew to prevent mechanical shearing [9, 57]. The mature root cells and hypocotyls of *tno1* seedlings treated with PPD showed marked disruption of cell morphology, suggesting a role for TNO1 in the cell maturation or expansion process. This model would also explain why *tno1* roots skew despite the transverse MT arrays in the epidermal cells of the root elongation zone. Vertically-grown *tno1* seedlings have similar root lengths to WT seedlings, suggesting normal cell expansion during vertical growth. Thus TNO1 might be required specifically for cell expansion during deviation of roots from their growth trajectory.

During cell expansion and cellulose deposition, the cellulose synthase complex traffics via the TGN [60, 61]. The observed defects in cell morphology and expansion could be due to the role of TNO1 in proper localization of the TGN-localized Q-SNARE SYP61 [31, 32]. Proteomic analysis of SYP61-containing vesicles [34] revealed the presence of cellulose synthase complex subunits, suggesting a role in trafficking of cellulose synthase subunits, which then drives cellulose deposition. MT array dynamics modulate the deposition of cellulose microfibrils in expanding cells since cellulose synthase runs parallel to the MT array, with CELLULOSE SYNTHASE INTERACTIVE1 acting as a link between the cellulose synthase machinery and cortical MTs [62–65]. MTs also regulate exocytosis of vesicles containing cellulose synthase complex subunits or complex-associated proteins such as KOR1 and CSI1 [66–68]. Disrupting MT arrays therefore affects cellulose synthesis, deposition and orientation, while conversely, inhibiting cellulose synthase activity causes defects in MT arrays [69, 70], which can potentially affect directional growth patterns [18]. Inhibitors of cellulose synthase trafficking such as CESTRIN cause MT instability and reduced cellulose content, and increase SYP61 and cellulose synthase co-localization [68]. Since CESTRIN does not affect the bulk secretory and endocytic routes, the SYP61-decorated domain of the TGN and its associated proteins such as TNO1 seem to be crucial for cellulose deposition and, indirectly, MT stability. However, mutations in cellulose synthase components (*rsw1–1*, *any1*) or treatment with cellulose synthase inhibitors does not induce CFR [58, 71, 72]. Moreover, Sugimoto (2003) also reports that a cellulose synthase mutant can undergo CFR even when the MTs are compromised [58].

Trafficking of other proteins required for cell expansion and root movement might also be disrupted in *tno1* mutants. For example, SKU5 is required for enzymatic reactions at the cell wall, and mutants lacking this protein show enhanced root skewing possibly due to altered cell wall composition during cell expansion [20]. Our observations suggest that CFR formation involves complex interactions and is not simply dependent on MT array and cellulose deposition patterns. Current models of the mechanisms of helical growth have been challenged by studies on *tortifolia2* mutants, in which individual freely growing trichomes twist. These mutants therefore uncouple cell division patterns and tissue strain from twisting and suggest that individual twisting of cells can translate to higher-order organ twisting [73]. Understanding the exact role of TNO1 at the TGN and the mechanism by which it modulates CFR and skewing will thus be a challenge.

Our results suggest that TNO1 and the TGN play a key role in CFR and root movement, adding another component to the network controlling root movement. Further experiments are necessary to test the exact role of TNO1 and its interacting partners in regulating root skewing and CFR formation. Future studies investigating TNO1’s role in cellulose synthase trafficking, MT stability and cell expansion dynamics in skewing roots will increase our understanding of the function of TNO1. Since TNO1 affects auxin transport [36], our study now points to the possibility of an impact of TNO1 on auxin transport, MT dynamics and cell wall trafficking at the TGN. Discovering TNO1’s precise role will help in elucidation of the function of the TGN SNARE machinery in plant growth and morphogenesis.

## Conclusions

In conclusion, we have discovered a role for a TGN-localized protein in root skewing on the surface of impenetrable media. This adds to the growing list of endogenous factors that aid root movement. Our data, alongside prior investigations, also suggest a possible link between TNO1, auxin-dependent and MT-associated processes. A better understanding of these mechanisms can lead to information that can be used to manipulate root growth and development patterns in various soil conditions and improve overall root architecture. This could subsequently result in engineering superior crops with better soil penetration and nutrient assimilation traits.

## Abbreviations

CFR: Cell file rotation
MT(s): Microtubule(s)
PPD: Propyzamide
SNARE: Soluble *N*-ethylmaleimide–sensitive factor attachment protein receptor
TGN: *Trans-*Golgi network
TNO1: TGN-localized SYP41-interacting protein
